# Molecular characterization of multi drug resistant *Escherichia coli* isolates at a tertiary hospital in Abuja, Nigeria

**DOI:** 10.1038/s41598-022-19289-z

**Published:** 2022-09-01

**Authors:** Nubwa Medugu, Mabel Kamweli Aworh, Kenneth Iregbu, Philip Nwajiobi-Princewill, Khadija Abdulraheem, Dawn M. Hull, Lyndy Harden, Pallavi Singh, Stephen Obaro, Abiodun Egwuenu, Siddhartha Thakur

**Affiliations:** 1 Department of Medical Microbiology, National Hospital Abuja, Abuja, Nigeria; 2 Department of Veterinary and Pest Control Services, Federal Ministry of Agriculture and Rural Development, Abuja, Nigeria; 3 Department of Population Health and Pathobiology, College of Veterinary Medicine, North Carolina State University, Raleigh, NC USA; 4 Northern Illinois University, De Kalb, IL USA; 5 University of Nebraska Medical Center, Omaha, USA; 6 Nigeria Center for Disease Control, Abuja, Nigeria

**Keywords:** Biological techniques, Genetics, Molecular biology, Diseases, Medical research

## Abstract

Infections caused by multi-drug resistant *Escherichia coli* cause significant morbidity and mortality especially in developing countries. In this study, we describe the molecular characteristics of *E. coli* isolated from clinical specimens and the patients’ outcomes. Phenotypic methods were used in the identification and antimicrobial susceptibility testing of *E. coli* from clinical specimens from a tertiary hospital in Abuja, Nigeria. Whole genome sequencing was used to describe the antimicrobial resistance genes, serotypes, sequence types/clonal complexes, and mobile genetic elements. The mean age of the patients was 20.3 years with 70.1% females and majority of isolates 75% from urine, 21% from blood cultures, and 3% each from cerebrospinal fluid and endo-cervical swabs. Of the 107 non-duplicate *E. coli* isolates, 101 (94.3%) were resistant to ampicillin, 95 (88.8%) to trimethoprim/sulfamethoxazole, 86 (80.4%) to ceftriaxone, 60 (56.1%) to gentamicin, and eight (7.5%) to meropenem. There were 102 (95.3%) isolates that were multi-drug resistant (MDR). Expression of Extended Spectrum Beta Lactamase (ESBL) phenotype was detected in 54 (50%) and *bla*_CTX-M-15_ genes detected in 75 (70.1%) isolates. The carbapenemase genes *bla*_NDM-1_ and *bla*_NDM-5_ were detected in six (5.6%), while the AmpC gene- *bla*_CMY-2_, was detected in seven (6.5%) isolates. Two (1.9%) isolates simultaneously harboured the *bla*_OXA-1_*, bla*_CMY-2_*, bla*_CTX-M-15_, and *bla*_NDM-5_ genes. In total, 35 sequence types (STs) were found with the majority being ST131 (n = 23; 21.5%). The most common serotype was O25:H4 associated with all 23 strains of ST131, followed by O1:H6/ST648 (n = 6). The ST410, ST671, and ST101 strains displayed phenotypic resistance to wide array of antibiotic classes and harbored high numbers of antibiotic resistance genes via in-silico analysis. The ST410 strain in particular harbored a higher number of antibiotic resistance genes and was phenotypically resistant to a wider array of antibiotics. Four pairs of isolates were closely related with three isolates (ST131, ST38, ST652) having a pairwise SNP difference of zero. 71/72 75/76 52/14. The MDR *E. coli* lineages circulating in this setting pose a clinical and public health threat as they can hinder effective prevention and management of infections. The genetic diversity and MDR *E. coli* with the emergence of ST410 and ST101 clones is concerning because of the potential for rapid dissemination in hospitals and communities- further increasing the problems of antibiotic resistance. Continuous routine surveillance of *E. coli* infections for AMR in hospitals becomes imperative, aimed at development of effective antimicrobial stewardship programs, facilitating prudent use of antimicrobial agents, and limiting dissemination of resistant strains.

## Introduction

One of the greatest threats to global health in the twenty-first century is antimicrobial resistance (AMR)^1^. AMR is both a one-health concept and a one-world problem spreading globally across humans, and animals^2^. Serious concern exists about the impending inability to treat common bacterial infections due to increasing rates of AMR across both humans and animals^3^. *Escherichia coli* has been implicated in many infections with increasing reports of resistance to commonly used antibiotics^4–6^. *E. coli* is one of the most common causes of sepsis and urinary tract infections (UTI) and we noted the high rates of resistance among this group of isolates. Infections with *E. coli* have been reported to be associated with increased length of hospital stay, higher cost of care, drain on limited resources, and high rates of morbidity and mortality^7^. Myriad factors drive AMR with increased/inappropriate use of antimicrobials for prophylaxis, treatment and animal growth promotion being primary driving forces^8,9^. In developing economies of sub-Saharan Africa (SSA) where the AMR burden is already reported as high^10,11^, inadequate environmental hygiene, poverty, poor healthcare systems, antibiotic-laden animal feeds, fake/sub-standard antimicrobials, with a background of expensive second‐line treatments, potentially create conditions for AMR pathogens to emerge and thrive^12,13^. This problem, in addition to the high burden of infectious diseases, fewer antibiotics in the market, and poor laboratory diagnostics, makes for a “perfect storm” for the emergence and spread of resistant bacterial strains.

*Escherichia coli* is an important pathogen causing infections ranging from mild to life-threatening with increasing reports of morbidity and mortality due to AMR *E. coli*^3,14^. Detailed characterization of molecular and clinical epidemiology of *E. coli* in Nigeria is sparse. This information is vital to understanding the strains in circulation in this population—a key step in designing impactful control measures. In this study, we aimed to characterize the *E. coli* strains in our archive to define the clinical conditions they are associated with and understand the patterns of antibiotic resistance. We aimed to also decipher using in silico methods, the serotypes, sequence types, and clonal complexes while understanding the antimicrobial resistance genes and suggest ways to combat and control the contemporary outlook of infections caused by this pathogen.

## Results

### Patient characteristics and outcomes

**Figure 1:**
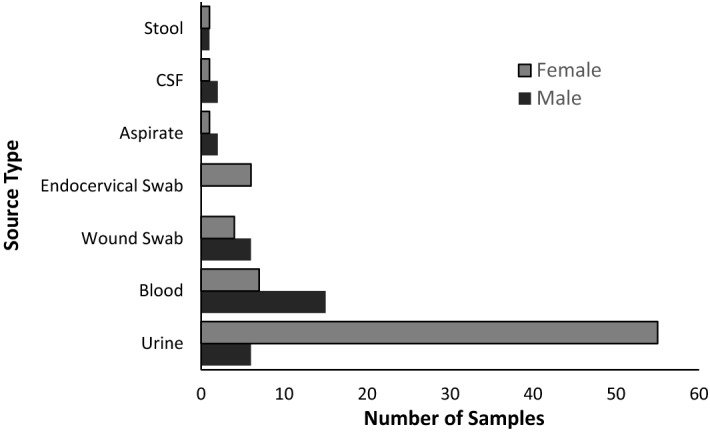
Gender-related occurrence of *E. coli* infection among hospital patients.

The mean age of the patients was 20.3 ± 20.6 years, gender pattern of 75 (70.1%) females and 32 (29.9%) males. Majority of isolates were cultured from urine 61 (57%) and blood culture 22 (20.5%) and least from CSF and ECS with 3 (2.8%) each (Fig. 1).

Over half of the patients 53% (n = 62) had been on antibiotics within the week prior to sample collection. The most common antibiotic patients had used was ceftriaxone 43.9% (n = 47), followed by amoxicillin-clavulanate 15.9% (n = 17) and gentamicin 14.0% (n = 15). Six patients (5.6%) were treated with both ceftriaxone and ciprofloxacin. Fifty-two (48.6%) of the patients were hospitalized at the time of sample collection. There was sufficient data to definitively categorize 75 (71.4%) of patients in this study as having hospital or community acquired infections. Of these 75 patients with relevant data, seven (9.3%) potentially acquired the infection from the hospital and 68 (90.7%) of the cases were potentially acquired from the community. Nine of the ill patients possessed additional risk factors for development of severe infections such as diabetes (n = 4), prematurity and low birth weight (n = 3), cancer of the prostate with chronic pyelonephritis (n = 1), and severe burns with pneumonitis (n = 1). Patient outcome at 30–60 days were available for 86 patients with a total of five mortalities- four from sepsis (isolates from blood culture) and one from meningitis (isolate from CSF). For the mortalities, the average length of stay in the hospital was 16.8 days (range 4–33 days). AMR genes carried on the isolates ranged from 7 to 17 (Supplementary table S2). For twenty-one patients, follow-up data was not available.

### Phenotypic antimicrobial resistance profile

Antimicrobial susceptibility testing detected resistance to ampicillin 94.4% (n = 101), ceftriaxone in 80.4% (n = 86) and meropenem in 7.5% (n = 8). Of the 107 *E. coli* isolates, 96.3% (n = 103) were multidrug resistant (MDR) (Table 1). Three isolates (NHA004, NHA029, and NHA040) were resistant to all antibiotics tested. Majority of isolates (n = 90, 84.1%) were resistant to drugs across at least five antibiotic classes.

**Table 1:** Antimicrobial resistance profiles of clinical *E. coli* isolates recovered from patients.

Drug class	Drug	MIC Resistance breakpoint µg/mL	Susceptible n (%)	Intermediate n (%)	Resistant n (%)
Penicillins	Ampicillin (AMP)	> 32	6 (5.6)	–	101 (94.4)
Tetracyclines	Tetracycline (TET)	> 16	8 (7.5)	–	99 (92.5)
Folate Pathway antagonists	Sulfisoxazole (FIS)	> 512	7 (6.5)	3 (2.8)	97 (90.7)
	Trimethoprim/sulfamethoxazole (TMP/SMX)	> 4/76	12 (11.2)	–	95 (88.8)
Quinolones	Ciprofloxacin (CIP)	> 1	15 (14.0)	2 (2.0)	90 (84.1)
	Nalidixic acid	> 32	9 (8.4)		98 (91.6)
Aminoglycosides	Gentamicin (GEN)	> 16	47 (43.9)	–	60 (56.1)
Phenicols	Chloramphenicol (CHL)	> 32	64 (59.8)	14 (13.1)	29 (27.1)
B-lactam inhibitors	Amoxicillin-clavulanate (AMC)	> 32/16	37 (34.6)	39 (36.5)	31 (29.0)
Cephem	Ceftriaxone (CRO)	> 4	21 (19.6)	–	86 (80.4)
	Cefoxitin (FOX)	> 32	80 (74.8)	8 (7.5)	19 (17.8)
Carbapenems	Meropenem (MEM)	> 4	99 (92.5)		8 (7.5)
Resistance to 3 or more classes of antibiotics	MDR	n/a	–	–	102 (95.3)

### Detection of ESBL-producing *E. coli* isolates

**Figure 2:**
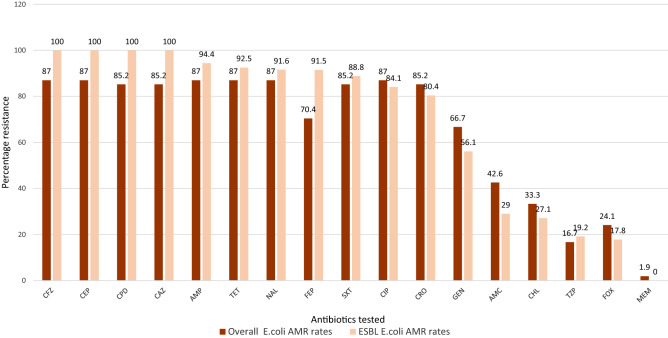
Comparison of antimicrobial resistance profiles of all isolates vs ESBL *E. coli* isolates expressed in percentages.

Approximately half of the isolates, 50% (n = 54) expressed an ESBL phenotype. Of these, 87.0% (47/54) were confirmed to be ESBLs. Figure 2 shows comparison between the AMR rates of ESBL isolates which are higher than those of the non-ESBL strains across most antibiotic classes. The ESBL positive strains exhibited 100% resistance to cefazolin, cephalothin, cefpodoxime, and ceftazidime. The *E.coli* strains showed low resistance rates to imipenem and meropenem with (1/54; 1.9%).

### Genetic characterization of ESBL and carbapenemase producing *E. coli* isolates

**Figure 3:**
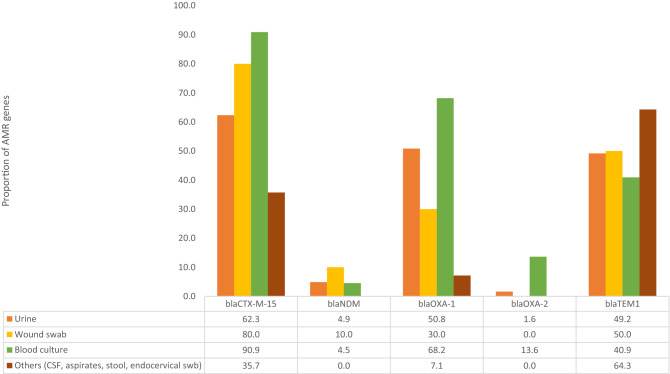
Comparison of ESBL and Carbapenem AMR genes distribution based on clinical specimens expressed in proportions.

The *bla*_CTX-M-15_ gene was the most identified ESBL gene in 75 (70.1%) isolates. This was followed by *bla*_CTX-M-27_ gene in four (3.7%); *bla*_CTX-M-14_ gene in three (2.8%) and *bla*_CTX-M-65_ gene in two (1.9%) isolates. The *bla*_OXA-1_ and *bla*_OXA-2_ genes were detected in 50 (46.7%) and four (3.7%) of the isolates, respectively. The carbapenemase genes *bla*_NDM-1_ and *bla*_NDM-5_ genes were detected in two (1.9%) and four (3.7%) of the isolates respectively. The plasmid-encoded *AmpC* gene; *bla*_CMY-2_, was detected in seven (6.5%) isolates. Two (1.9%) isolates simultaneously harbored the *bla*_OXA-1_, *bla*_CMY-2_*, bla*_CTX-M-15_, and *bla*_NDM-5_ genes while one (0.9%) isolate harbored both *bla*_CTX-M-14_ and *bla*_NDM-5_ genes. One (0.9%) isolate co-harbored *bla*_NDM-1_ and *bla*_OXA-2_. Thirty (28%) isolates co-harbored *bla*_CTXM-15_ *and bla*_TEM-1_ while 17 (15.9%) had *bla*_CTXM-15_ and *bla*_OXA-1_*.* When the beta-lactamase genes were analyzed according to infection source, the *bla*_CTXM-15 and_ *bla*_OXA-1_ were predominant among blood specimens while *bla*_NDM_ were predominant among wound swab specimens as shown in Fig. 3.

### Resistance genes detected in *E. coli* isolates

Fifty-seven different antibiotic resistance genes were identified from strains in this study (Table 2). β-lactam resistance genes accounted for most observed resistance genes with 13 different variants including the classical ESBL *bla*_CTX-M_ type (7 types), and *AmpC* producing *bla*_CMY_ type (1). Other β-lactam genes detected included *bla*_OXA_ (2) and bla_TEM_ (2). This is further detailed in Table 2. Resistance to aminoglycosides was mediated by ten genes out of which *aac(3*′*)-IIa* was the most detected in 29.9% of isolates. In similarly high prevalence at 29% (n = 31) was the *aac(3)-Iie,* a major aminoglycoside-modifying enzyme. We found genes conferring erythromycin, macrolide, phenicol, tetracycline, sulfonamide, and trimethoprim resistance (Table 2). This study also detected *bla*_NDM-1_ and *bla*_NDM-5_ which confer carbapenem resistance as well as *fosA3* and *fosA7* conferring fosfomycin resistance. The highest number of antibiotic resistance genes detected in a single isolate (n = 22) was detected in a strain (NHA040) originating from a blood culture.

**Table 2:** Acquired antibiotic resistance genes detected in *E. coli* isolates recovered from patients attending the National Hospital Abuja, Nigeria expressed in frequencies.

Antibiotic class^a^	Resistance determinants of *E. coli* isolates (no of isolates)^b^
Aminoglycoside	*aac(3)-IId (25), aac(3)-IIe (31)*, *aac(6*′*)-Ib-cr (47)*, *aad*A5 (57), ant(2′′)-Ia (2), *aph(3*′′*)-Ib (60)*, *aph(3*′′*)-VI (1), aph(6)-Id* (61)
β-Lactam	*bla*_CTX-M-14_ (3), *bla*_CTX-M-15_ (71), *bla*_CTX-M-27_ (3), *bla*_CTX-M-55_ (1), *bla*_CTX-M-65_ (1), *bla*_CTX-M-130_ (1), *bla*_CTX-M-176_ (1), *bla*_OXA-1_ (50), *bla*_OXA-2_ (4), bla_TEM-1_(53), bla_TEM-190_(1), *bla*_CMY-2_ (7)
Carbapenem	*bla*_NDM-1_ (2), *bla*_NDM-5_ (4)
Erythromycin	*ermB* (11) *ermD* (2)
Fosfomycin	*fosA3* (1) *fosA7* (1)
Macrolide	*mdf*A (101), *mph*A (69)
Phenicol	*catA1* (23), *catA2* (3), *catB3* (48), *cmlA1* (4)
Sulfonamide	*sul1* (67), *sul2* (52), *sul3* (5)
Tetracycline	*tet*A (53), *tet*B (78), *tet*M (1)
Trimethoprim	*dfrA1* (3), *dfrA7* (2), *dfrA8* (1), *dfrA12* (11), *dfrA14* (10), *dfrA17* (53), *dfrA82* (2), *dfrB4* (1)
Quinolone	*qepA* (3), *qepA1* (3), *qepA2* (1), *qepA4* (5), *qnrB19* (1), *qnrS1* (8), *qacE* (1)
Multi drug class gene—phenicols, lincosamids	*mdtM* (94), *emrD* (2)

^a^Drugs corresponding to each antibiotic class used in our study are as follows: aminoglycosides, streptomycin, gentamicin; beta-lactams, ampicillin, ceftriaxone, cefotaxime, ceftazidime, ceftiofur, cefoxitin, amoxicillin-clavulanic acid; phenicols, chloramphenicol; folate pathway antagonists, trimethoprim-sulfamethoxazole, sulfisoxazole; tetracycline.

^b^The numbers of isolates carrying each resistance determinant are presented in parentheses.

### Mobile genetic elements

Many of the AMR genes detected in this study were located on insertion sequences. There was an average of five insertion sequences containing AMR genes per isolate (range of 0–8 insertion sequences per isolate). A total of 492 plasmids were detected in the analysed strains with the most prevalent being IncFI (n = 318; 64.6%), Col (n = 49; 10.0%), IncY (n = 27; 5.5%), IncQ1 (n = 26; 5.3%), and IncB/O/K/Z (n = 25; 5.1%). Less common plasmids consisted of IncI (n = 18; 3.7%), IncN (n = 17; 3.5%), IncX (n = 7; 1.4%), IncR (n = 4; 0.8%), and IncC (n = 1; 0.2%). In 19 isolates, there was association of ARGs with the plasmid replicons. The IncFll plasmid carrying *bla*_TEM-1b_ was found in three isolates with its variants also found carrying resistance genes as follows: IncFll (Prsv107) harboring *bla*_TEM-1b_ (n = 3), IncFll (Prsv109) harboring *bla*_TEM-1b_ (n = 1), IncFll (Prsv107) harboring *bla*_TEM-1d_ (n = 1), IncFll (pama1167) harboring both *bla*_EM-1d_ (n = 1) and *bla*_NDM-5_. The IncQ1 plasmid carrying *sul2* was found in ten isolates. The plasmid IncB/O was found carrying one *bla*_CTX-M-14_.

### Serotyping and multi-locus sequence types of *E. coli* isolates

There were 21 H types across all 107 isolates with the most common being H4 (n = 30, 28.0%), H9 (n = 25, 23.4%), H6 (n = 11, 10.3%) and H21 (n = 6, 5.6%). There were 25 different O types across the samples with O target not detected in 16 isolates. The common O groups detected are O25 (24, 22.4%), O101 (12, 11.2%), O1 (7, 6.54), and O9 (8, 7.5%).

The 107 *E. coli* strains were associated with 35 different sequence types, the most common was ST131 that belongs to CC131, n = 23 (21.5%). All 23 ST131 isolates belonged to serogroup O25:H4. The next most common was ST410 (14.0%) in CC23/26 (n = 15, 14.0%) which were in serogroup O8:H9 (5), O76:H9 (2), and O104:H9 (1). Seven ST410 were associated with H9 but had no corresponding O antigen target identified. Resistance to the highest number of antibiotics was detected in ST671 (n = 5, 4.7%) and ST648 (n = 7, 6.5%). Also, with resistance to large numbers of antibiotics were ST410 and ST131. Five novel STs were detected with all being isolated from urine samples.

There was a high degree of association in *E. coli* isolates with the following serogroup/ST; O25:H4/ST131 (n = 23, 21.5%), O1:H6/ST648 (n = 6, 5.6%), O8:H9/ST410 (n = 5, 4.7%), and O188:H31/ST101 (n = 4, 3.7%) (Supplementary table 1).

### SNP based phylogenetic assessment of *E. coli* strains

**Figure 4:**
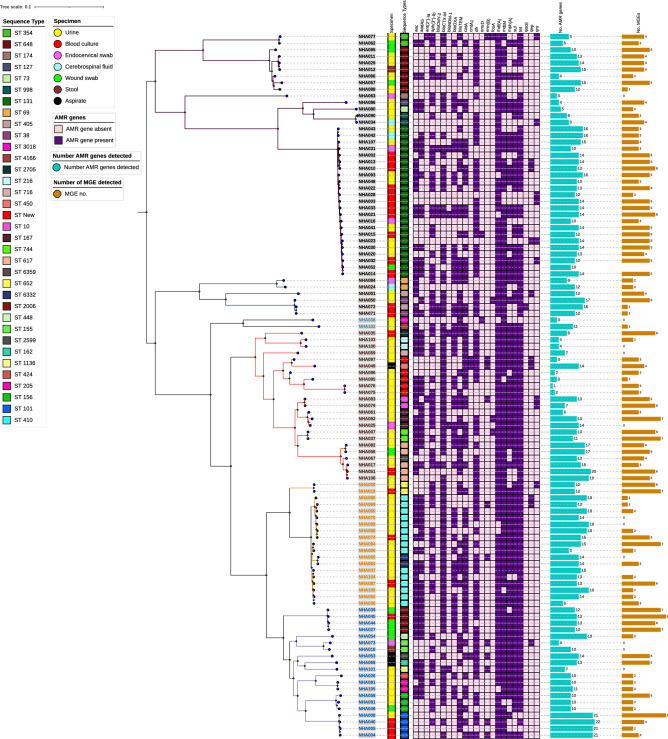
Phylogenetic analysis of SNPs from *E. coli *strains in study with colour strips of specimen source and sequence types, heat map of AMR genes, bar chart of number of resistance genes identified and bar chart of number of mobile genetic elements identified and bar chart of number of mobile genetic elements identified per isolate. Tree was midpoint rooted. The SNP was based on the reference strain NZ_CP028166.1

There was wide diversity of the strains causing infections in this population (Fig. 4). The phylogenetic tree and distance matrix shows close relationships between NHA052/NHA014 (ST 131), NHA071/NHA072 (ST38, ST405), and NHA076/NHA075 with pairwise distances of zero.

## Discussion

In this study, we investigated the characteristics of *E. coli* isolates recovered from patient specimens in National Hospital Abuja between March 2019 and September 2020. We found a very high proportion of *E. coli* isolated from patients were MDR.

A large proportion of clinical specimens analyzed (53%) were collected from patients who were on antibiotics at the time specimens were collected. This high rate of antibiotic use has also been documented in similar settings within the region where rates of 40–80% were reported^15–17^. The high and increasing rates of antibiotic use in this region may be the primary driver of AMR due to selective pressure. Here, we found that ceftriaxone was the most frequently used antibiotic and not surprisingly, had very high resistance rate that may have contributed to high resistance observed in our study. High ceftriaxone use in hospitals and its increasing resistance rates have been reported in similar clinical settings in Africa and other developing countries with use reported as 51% in Tanzania and 59% in Ethiopia^18–20^. Similarly high resistance rate to ceftriaxone was found in a study on UTI in the southern part of Nigeria which reported 86%^21^. Ampicillin with an almost absolute resistance rate in this study, has thus been rendered almost impractical in this population although lower rates were found in a study targeting community infections in 2015^22^. Following good susceptibility profile of cefoxitin found in this study, further real life investigation into its potential usefulness in this population is warranted as it has proven to be a good resource on other settings^23^. Chloramphenicol displayed a relatively good profile with most isolates being susceptible. This is similar to reports from studies outside of the region^24^ and contrasts with other studies which reported higher resistance to chloramphenicol in Nigeria^25^. Despite known side effects, chloramphenicol preserves some functionality as shown in this study and warrants consideration in our setting because it is relatively cheap and available. In this study for instance, chloramphenicol was the only susceptible antibiotic in two patient cases while in 13 other cases, carbapenems were the only other available option. Unfortunately, the carbapenems are either unavailable or unaffordable for most of the Nigerian population. The MDR rates of 95% (including ESBL phenotype of 50%) was very high and could be attributable to tertiary hospital setting. The high MDR rates may could also be attributable to more purposive storage of resistant isolates in the biorepository, in which case further study will be required to determine the true rate. Most blood culture and urine isolates were resistant to the most used antibiotics including those recommended for empiric treatment (aminoglycosides and 3rd generation cephalosporins). The aminoglycoside gentamicin was in use for 14% of the patients in this study which possibly may contribute to the high resistant rates of 56% was found for gentamicin. A slightly lower gentamicin resistance of 44% was reported from another study in Nigeria published in 2019^26^. The scenario is compounded by limited number of therapeutic agents. Therefore, prudent use of the antibiotics become imperative. The implication is failed treatments, disease progression, and dissemination of the clones of concern.

There was wide array of ARGs in *E. coli* with some isolates harboring as many as 22 acquired AMR genes which is one of the highest reported worldwide^27^. While the *bla*_CTX-M-15_ is commonly detected in the region as described in other studies^28,29^, other uncommon β-lactamase genes such as the plasmid encoded *AmpC* gene *bla*_CMY-2,_ *bla*_CTX-M-65_ and *bla*_CTX-M-130_ were also detected in this study^14^. The *bla*_CMY-2_ as a plasmid encoded *AmpC* gene has rarely been reported in this region in molecular characterization of clinical *E. coli* strains. Interestingly, *bla*_SHV_ genes were not detected in these isolates unlike other studies^30^. The metallo-β-lactamase genes *bla*_NDM-1_ and *bla*_NDM-5_ which mediate resistance to carbapenems which are often regarded as last resort antibiotics were found at relatively lower rates of about 2% in another Nigerian study compared to the 7% reported in our study^30^.

All isolates had mobile genetic elements (plasmids and insertion sequences) carrying multiple resistance genes. Unfortunately, these serve as a pool for horizontal AMR transfer. The most prevalent plasmid in this study IncFII was also found predominating in other studies, although no other studies from SSA have reported this^31^.

In concordance with other studies, we similarly found ST131 was the more frequently seen ST in extra-intestinal infections^32^. The ST131 strain is associated with increased expression of virulence and beta-lactamase genes^33^. Of more concern in this population however were STs with high resistance rates such as the ST101 which are of great concern in this population. The second most common lineage was ST410 and in this study, it had higher rates of antibiotic resistance than ST131 and we postulate could be an epidemic clone. Other ST lineages we found with very high resistance rates and potentially emerging strains of concern are ST671 and ST648. The four pairs of isolates that seemed very closely related in pairwise SNP differences may be inferred to have been acquired from a common source and warrant further investigation into potential outbreak within the hospital.

Our inability to retrieve some patient clinical outcome data were some limitations we encountered.

## Conclusions

This study has described the clinical infections and poor outcomes of *E. coli* infections alongside detailed phenotypic and molecular characterization of resistance genes. The infections caused by *E. coli* as detected in this study caused significant morbidity and mortality and measures to reduce these should be implemented in the hospital. The observed widespread resistance across multiple antibiotic classes warrants modification of present empiric therapy within the facility and calls for more studies to determine if the empiric therapy modifications should be carried out in more centers. Of more concern in infection treatment in this populace beyond ST131 are ST410 and ST101 because of the higher phenotypic and molecular resistance markers they have. The novel and emerging STs in this populace along with especially high resistance rates are concerning because this potentially, is a pool from which global dissemination could occur.

Continuous surveillance of *E. coli* infections in Nigeria—the most populous country in Africa using conventional and WGS methods is warranted to understand development and transmission dynamics—these are key to creation and implementation of locally specific antibiotic stewardship and infection control strategies as it is obvious that a generic approach will be largely unsuccessful in Nigeria.

## Methods

### Study design and sample collection

This cross-sectional study was conducted at the National Hospital Abuja (NHA), a 425-bed tertiary facility that also provides primary and secondary health care. A total of 107 non-duplicate *E. coli* strains previously stored in 50% glycerol at − 80 °C were studied. The source of isolates was clinical specimens analyzed in the laboratory between March 2019 and September 2020. Sociodemographic and clinical data was retrieved from hospital records and all patient identifiers anonymized. The present study was approved by the National Hospital Abuja (NHA) ethics committee/institutional review board and was exempted from informed consent requirements owing to its retrospective design. The approval number of the study is NHA/EC/033/2018.

### Isolation, identification, and antibiotic susceptibility testing of* E. coli* isolates

Brilliance UTI medium and Cysteine lactose electrolyte deficient agar-CLED (*Oxoid*, Basingstoke, UK) were used for primary culture of urine. Primary blood culture was inoculated into BacTalert bottles with sampling based on weight-based criteria and incubated in the BacTalert system BacT/Alert system (BioMerieux, Marcy l′Etoile, France) until positive. Sheep blood agar, Chocolate agar, and MacConkey agar were used for other specimens as required. Culture plates were incubated for 18–24 h at 35.5 °C in ambient air (Chocolate agar incubated in 5% CO_2_).

The Vitek 2 Compact (bioMerieux, Marcy l'Etoile, France) with VITEK 2 GN ID card was used for bacterial identification and the VITEK 2 AST-280/ VITEK 2 AST-281 cards for susceptibility testing. Clinical and Laboratory Standards Institute (CLSI) M100 30th Edition guidelines^34^ was used for interpretation of MIC results. *E*. *coli* ATCC 25922 was used as control. Multi-drug resistance was defined as resistance to antibiotics in three of more different classes.

Shipped isolates to Thakur Molecular Epidemiology laboratory, North Carolina State University (NC State), USA underwent microdilution assay with the Gram-negative Sensititre™ (CMV3AGNF) plate (Trek Diagnostic Systems, OH). Plates were read using *Sensititre* ARIS and interpreted according to CLSI M100 28th Edition guidelines^35^. *E*. *coli* ATCC 25922 was used as a control.

### Detection of ESBL phenotype

*E. coli* isolates were screened for extended-spectrum beta-lactamase (ESBLs) using TREK *Sensititr*e ESBL Plate (Trek Diagnostic Systems, Thermo Fisher Scientific, Lenexa, KS, USA) and characterized as positive for ESBL if there was ≥ 3 two-fold concentration decrease in MIC for cefotaxime or ceftazidime with clavulanic acid compared to MIC for the respective antimicrobial agent tested alone^36^. Multidrug resistance (MDR) was defined as resistance to at least three classes of antimicrobials^37^.

### Whole-genome sequencing for detection and characterization of AMR genes, plasmids, and insertion sequences

Whole-genome sequencing (WGS) was performed for all isolates. Briefly, after culture on Luria–Bertani (LB) agar at 37 °C, genomic DNA was extracted using *Lucigen MasterPure* Gram Positive DNA Purification Kit. Libraries were prepared for each isolate using Nextera XT DNA Sample Prep Kit (Illumina Inc., San Diego, CA), and sequenced on an Illumina Miseq platform using a 2 × 250 paired-end approach (Illumina Inc., San Diego, CA). Raw sequencing reads were demultiplexed and converted to fastq files using CLC Genomics workbench version 9.4 (Qiagen bioinformatics, Valencia, CA). Raw reads were uploaded to the National Center for Biotechnology (NCBI) database Bio project ID number PRJNA293225. Prediction of AMR was conducted by using Mobile Element Finder v1.0.3 (2020-10-09) and selecting Acquired Antimicrobial Resistance genes (ResFinder)^38^. We located the mobile genetic elements (MGEs) associated with resistance genes by using Mobile Element Finder with database v1.0.2 (2020-06-09)^38^. Each resistance gene was classified as being carried by a plasmid, or MGE, or as not associated based on the MGE output. Plasmids were detected using PlasmidFinder-2.0 with threshold for minimum at 95% identity and minimum 60% coverage using draft genome assemblies^39^. The high-quality Illumina paired-end reads generated were assembled de novo into the draft genome sequence for every isolate using SPAdes assembler v.3.13.1. Quality assessment for genome assemblies was carried out using QUAST^40^.

### Multi-locus sequence typing (MLST) and serotyping

In silico typing with regard to O:H serotypes based on WGS of assembled genomes/contigs was carried out by using Serotype Finder 2.0^41^ with selected threshold of 90% identity and 60% total serotype gene length. In silico MLST-analyses using previously described seven housekeeping genes (*adk, fumC, gyrB, icd, mdh, purA, and recA)*^42^ was performed. PubMLST—Achtman scheme was performed to identify the sequence types (STs) and clonal complexes (CCs)^43^. Isolates with 100% match against known MLST alleles were assigned STs and CCs. Those without perfectly matching alleles were identified as unknown STs^44^.

### Calling SNPs and inferring Phylogeny

The FASTA files generated from WGS were uploaded unto the CSI Phylogeny 4.1 service of Centre for Genomic Epidemiology (https://cge.cbs.dtu.dk/services/CSIPhylogeny/). CSI Phylogeny outputs were generated based on a selected reference sequence (*E. coli* NZ_CP028166.1) and downloaded as Newick and text files. Thresholds for SNP calling were for depth = 10×, for SNP quality − 30, for map quality − 25, and 1.96 for minimum Z score^45^. Visualization annotation, and management of tree files were performed using the interactive Tree of Life tool—iTOL v6 (http://itol.embl.de/itol.cgi). Pairwise SNP differences between genomes were computed to determine if isolates of different origins were related with SNP distances < 21 indicating close relatedness and 21–50 indicating some more distant relatedness^46^.

### Data collection and analyses

Information entered in MS Excel was analyzed using STATA (StataCorp. 2019. TX: StataCorp LLC). Data for phenotypic and genotypic characteristics were analyzed by computing frequencies and proportions. Means was calculated for age. Data relevant to this study are within the paper and available as supporting information.

### Ethics approval and consent to participate

The ethics review board of NHA reviewed and gave approval for the study (Approval number NHA/EC/033/2018). Two stage data encryption was done to ensure confidentiality and patient anonymity was assured. Only the principal investigator and KA had access to the patient details. All bacteria isolates used here were recovered from submitted clinical specimens at the NHA. The confidentiality of the information obtained was assured as all patient identifiers were anonymized. All procedures we performed were in accordance with the guidelines and regulations of the ethics review board.

## Supplementary Information

Supplementary Information.

## Data Availability

The datasets used and analyzed during this study are available from the corresponding author on request. Data used for this manuscript are included in this published article and its supplementary information files. The datasets generated during the present study are also available in the National Center for Biotechnology Information (NCBI) repository under the Genome Trakr project with the accession number PRJNA293225.

## Abbreviations

AMR: Antimicrobial resistance
ATCC: American type culture collection
CLSI: Clinical and Laboratory Standards Institute
ESBL: Extended-Spectrum Beta-Lactamase
MDR: Multi-drug Resistance
MIC: Minimum Inhibitory Concentration
MLST: Multi-locus Sequence Typing
NCBI: National Center for Biotechnology Information
SRA: Sequence Read Archive
ST: Sequence Type
WGS: Whole-genome Sequencing
